# Unraveling the role of peroxisome proliferator-activated receptor-β/δ (PPARβ/δ) expression in colon carcinogenesis

**DOI:** 10.1038/s41698-019-0098-x

**Published:** 2019-10-07

**Authors:** Jeffrey M. Peters, Vonn Walter, Andrew D. Patterson, Frank J. Gonzalez

**Affiliations:** 1 Department of Veterinary and Biomedical Sciences, The Center of Molecular Toxicology and Carcinogenesis, The Pennsylvania State University, University Park, State College, PA 16801 USA; 2 Departments of Public Health Sciences and Biochemistry, The Pennsylvania State University, College of Medicine, Hershey, State College, PA 16801 USA; 3 Laboratory of Metabolism, National Cancer Institute, Bethesda, MD USA

**Keywords:** Cancer, Colon cancer

## Abstract

The peroxisome proliferator-activated-β/δ (PPARβ/δ) was identified in 1994, but not until 1999 was PPARβ/δ suggested to be involved in carcinogenesis. Initially, it was hypothesized that expression of PPARβ/δ was increased during colon cancer progression, which led to increased transcription of yet-to-be confirmed target genes that promote cell proliferation and tumorigenesis. It was also hypothesized at this time that lipid-metabolizing enzymes generated lipid metabolites that served as ligands for PPARβ/δ. These hypothetical mechanisms were attractive because they potentially explained how non-steroidal anti-inflammatory drugs inhibited tumorigenesis by potentially limiting the concentration of endogenous PPARβ/δ ligands that could activate this receptor that was increased in cancer cells. However, during the last 20 years, considerable research was undertaken describing expression of PPARβ/δ in normal and cancer cells that has led to a significant impact on the mechanisms by which PPARβ/δ functions in carcinogenesis. Whereas results from earlier studies led to much uncertainty about the role of PPARβ/δ in cancer, more recent analyses of large databases have revealed a more consistent understanding. The focus of this review is on the fundamental level of PPARβ/δ expression in normal tissues and cancerous tissue as described by studies during the past two decades and what has been delineated during this timeframe about how PPARβ/δ expression influences carcinogenesis, with an emphasis on colon cancer.

## Introduction

The mouse and human peroxisome proliferator-activated-β/δ (PPARβ/δ, also referred to as PPARβ or PPARδ) were identified in 1994 and 1995, respectively.^1,2^ PPARβ/δ belongs to the nuclear hormone receptor superfamily. Nuclear hormone receptors are critical in the maintenance of cellular homeostasis by modulating expression of genes in response to ligand activation. For example, PPARβ/δ regulates skeletal muscle fatty-acid catabolism by regulating expression of genes that facilitate fatty acid transport across membranes and fatty acid catabolism in this tissue. (reviewed in ref. ^3^) Through this mechanism, muscle cells are able to survive during periods of starvation, which is the predominant state most organisms are in during a 24-hour period but can be fine-tuned because of this well-conserved pathway that responds to changes in cellular production of endogenous PPARβ/δ ligands. The release and transport of fatty acids into cells allows for increased levels of fatty acids and fatty-acid metabolites that can act as ligands for PPARβ/δ, thereby activating PPARβ/δ-dependent transcription of target genes that collectively promote fatty acid catabolism and the generation of intracellular adenosine triphosphate, thus providing cells with an energy source. There are many other examples of similar regulatory mechanisms for other nuclear receptors; cells respond to stimuli that most commonly include the presence of endogenous ligands and altered expression of critical proteins that maintain homeostasis.

Although early studies suggested that nuclear receptor signaling occurred through a static mechanism, it is now clear that nuclear receptors such as PPARβ/δ regulate transcription by a dynamic mechanism. (reviewed in ref. ^4^) There are several levels of regulation that modulate the activity of a nuclear receptor such as PPARβ/δ: (1) the relative level of receptor expression, (2) the relative concentration of available ligands, and (3) the availability of binding sites on chromatin. The relative expression of a nuclear receptor can be variable and there are a number of regulatory transcription factors that in turn modulate expression of nuclear receptors. Endogenous ligands are derived from metabolic “release” from sources such as lipids found in cell membranes, metabolism of various substrates, dietary intake, etc., and their fluctuation during a 24-hour period of time can in turn alter the activity of a given nuclear receptor. There are numerous enzymes that modify chromatin that are in constant flux and through their actions on chromatin, make binding sites more or less available for nuclear receptors. At last there is limited evidence that post-translational modifications of PPARβ/δ may impact its transcriptional activity. For example, results from one study suggest that PPARβ/δ can be de-SUMOylated and this may enhance expression of genes involved in skeletal muscle fatty oxidation.^5^ In addition, one study suggests that PPARβ/δ ligands may prevent degradation of PPARβ/δ-prolonging activity,^6^ however, this is not observed when expression of PPARβ/δ is not forced as typically found in normal cells.^7^ Thus, there remains a need to delineate whether post-translational modifications of PPARβ/δ impact receptor activity.

PPARβ/δ regulates many physiological processes. This includes increasing serum high-density lipoprotein,^8^ catabolism of fatty acids in skeletal muscle,^3^ regulation of serum glucose concentration,^9^ and terminal differentiation of a variety of cell types.^10^ Because of these important physiological roles, there remains strong interest in targeting PPARβ/δ for the treatment and prevention of diseases including atherosclerosis, diabetes, and obesity (reviewed in refs ^11,12^). Although the majority of these effects have been elucidated through the use of synthetic ligands for PPARβ/δ, it is important to note that the relative expression levels of PPARβ/δ is also an important feature that is required to mediate these important functions. Given the essential role of PPARβ/δ in normal physiology, it is interesting to note that the potential function of PPARβ/δ in carcinogenesis remains an area of controversy. This is owing to conflicting reports with some studies, suggesting that PPARβ/δ promotes cancer, whereas other studies indicate that PPARβ/δ inhibits cancer. The focus of the remaining review is on one fundamental level of regulation: expression of PPARβ/δ in colon cancer models, and what has been learned in the past 20 years about PPARβ/δ in colon cancer based solely on its relative expression.

## Physiological expression levels of PPARβ/δ in normal tissues

Basal expression of PPARβ/δ in normal tissues was examined at both the mRNA and protein levels in both human and rodent species. However, the scientific rigor and reproducibility of these assessments are quite variable. One of the first studies to examine expression of *Pparb/d* mRNA in different tissues used a northern blotting technique and samples from adult male rats.^2^ Results from these analyses suggested that expression of *Pparb/d* mRNA was relatively high in adrenal gland, heart, and intestine, moderately high in the brain, kidney, and spleen, and relatively low in the liver and testis. In this study, only a single sample from each tissue was examined in this study and no quantification was performed. Using in situ hybridization and immunohistochemistry, it was later suggested that *Pparb/d* mRNA was expressed in many tissues including hepatocytes, spleen, kidney, gastrointestinal (GI) tract and the brain in adult rats.^13^ Interestingly, in this study, the authors indicated that expression of *Pparb/d* mRNA was high in the hepatocytes, spleen, kidney and upper GI tract but lower in rat colon as compared with the small intestine. Although these analyses also included assessment of protein expression using a single antibody coupled with immunohistochemistry (IHC), it is difficult to determine the quantitative nature of these collective studies because details of the number of biological replicates, whether the samples were blinded by the investigators, and statistical analyses were not provided.^13^ Others examined basal expression of *Pparb/d* mRNA using an RNase protection assay in adult rats in fed and fasted states and revealed that the relative basal expression of *Pparb/d* mRNA was highest in the GI tract including both the small and large intestine, kidney, heart, diaphragm, esophagus, and liver.^14^ Basal expression of *Pparb/d* mRNA was also detected in the brain, tongue, lung, thymus, spleen, pancreas, adrenal gland, skeletal muscle and bladder as well, but expression was considerably lower as compared with the aforementioned tissues. Interestingly, the relative expression of *Pparb/d* mRNA was higher in fed rats as compared with fasted rats in the liver and kidney only suggesting a role for PPARβ/δ in these tissues during periods of starvation/feeding. Although no statistical analysis of the basal expression of *Pparb/d* mRNA was performed in these studies, the use of the sensitive RNase protection assay in groups of three to five animals yielded results that provided some of the strongest data at the time with respect to relative expression of *Pparb/d* mRNA in specific tissues in male rats.^14^ Another group examined *Pparb/d* mRNA in 39 different tissues from six C57BL/6 or Sv/129 mice using quantitative real-time polymerase chain reaction (qPCR).^15^ The analyses were focused on male mice with the exception of uterus, which was obtained from female mice. These results were fairly consistent with the data observed in male rats,^14^ with markedly high expression of *Pparb/d* mRNA being observed in colon, small intestine, and kidney, and relatively high expression in all other tissues examined.^15^ The latter included adrenal gland, skin, gall bladder, liver, heart, and thyroid gland. Importantly, expression of *Pparb/d* mRNA was noted in all 39 tissues and was not detected at low levels in any of the tissues examined.^15^ Collectively, the more rigorous studies performed in rodents to date are fairly consistent and show that expression of *Pparb/d* mRNA is relatively high in many tissues, in particular in the colon, small intestine, and kidney.

**Fig. 1:**
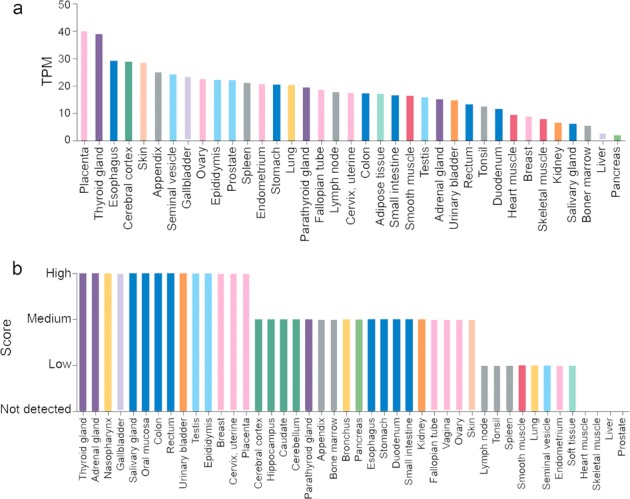
Relative expression of *PPARB/D* mRNA and protein in human tissues. **a** Relative *PPARB/D* mRNA expression in human tissues based on analyses of data from the Human Protein Atlas database (proteinatlas.org).^16–18,81^ **b** Relative expression of PPARβ/δ in human tissues based on analyses from the Human Protein Atlas database (proteinatlas.org).^16–18,81^ Relative expression of protein or mRNA based on analysis from the Human Protein Atlas on September 15, 2019, Version 19, Ensembl version 92.38

There are limited studies that have examined expression of *PPARB/D* mRNA in normal human tissues and the most comprehensive analyses to date are those deposited in the Human Protein Atlas (HPA; proteinatlas.org).^16–18^ For expression levels of *PPARB/D* mRNA in humans, the HPA contains data sets collected from three different sources: the HPA gene expression profile (37 tissues examined), the Genotype-Tissue Expression (GTEx) project (31 tissues examined), and the FANTOM project database (36 samples examined). Relative expression was determined by RNA-sequencing analyses for HPA and GTEx and the number of samples examined from each database is variable. Data are presented as transcripts per million and are a relative measure of the number of mRNA transcripts per gene in the tissue of interest. Similar to the data reported for rats and mice, expression of *PPARB/D* mRNA in humans is found in all tissues examined. Tissues with relatively higher mRNA transcripts include colon, small intestine, thyroid gland, skin, brain lung, kidney (GTEx was higher as compared with HPA), adipose, and adrenal gland.^16–18^ Thus, there is considerable overlap and consistency between rodents and humans in terms of relative expression of *PPARB/D* mRNA; notably tissues such as those that are epithelial in nature (colon, small intestine, liver, skin, etc.) and kidney and adrenal gland express appreciable levels of *PPARB/D* mRNA in humans (Fig. 1a), rats, and mice.

Expression of protein is more physiologically relevant as it is the protein that mediates the biological function of most gene products. By contrast to mRNA analyses, there are considerably fewer studies that have comprehensively examined protein expression quantitatively in either rodent or humans. The most extensive analyses of PPARβ/δ expression in mice was reported in 2008 and used a highly specific anti-PPARβ/δ polyclonal antibody.^19^ In male C57BL/6 mice, relative expression of PPARβ/δ was highest in small intestine, keratinocytes, colon, liver, kidney, and brain, but expression of PPARβ/δ was also high in heart, lung, skeletal muscle, spleen, and thymus.^19^ Most interestingly, expression of PPARβ/δ was predominantly higher in the nucleus as compared with cytosol in all tissues examined, suggesting that PPARβ/δ had a role in constitutive regulation of target gene expression.^19^ This was confirmed in part by demonstrating that PPARβ/δ could be co-immunoprecipitated with its heterodimerization partner retinoid X receptor. Moreover, another study demonstrated that expression of many genes was markedly increased or decreased by simply deleting expression of PPARβ/δ expression in mouse keratinocytes,^4^ a cell type known to express high levels of PPARβ/δ. This phenomenon was also observed in a human cell line in a separate study.^20^ Combined, these data suggest that PPARβ/δ is constitutively active in cells owing to the presence of endogenous ligands (agonists, antagonists, selective repressive ligands (inverse agonists)). This is consistent with what was also observed in cells with another PPAR isoform, PPARγ, in adipocytes.^21^ Expression of human PPARβ/δ protein has also been comprehensively examined by investigators as part of the HPA.^16–18^ Although the analyses of protein expression presented in the HPA is comprehensive in nature, it is limited in part owing to the reliance on IHC for assessment of relative expression of proteins, which has known limitations as discussed below. Given this weakness, it is important to note that there is validation of the antibodies used for the HPA, but that the validation is lacking a number of controls^22,23^ and as such these data are limited in rigor as well. In human tissues, relative expression of PPARβ/δ protein is very high to moderate in colon, skin, breast, thyroid gland, gall bladder, adrenal gland, brain, kidney, male and female reproductive tissues, and bone marrow (Fig. 1b). By contrast to male mice, relative expression of PPARβ/δ protein in humans is low in lung and spleen, and not detectable in liver, heart, and skeletal muscle (Fig. 1b). Combined, based on the expression patterns of both mRNA and more importantly protein, PPARβ/δ appears to have an important physiological role in epithelial cells such as colon, skin, lung, and liver, and molecular analyses suggest that this is likely owing to the presence of endogenous ligands that bind to and modulate the function of this receptor in multiple tissues.^4,24^

## Regulation of PPARβ/δ expression in colon cancer

The first study to suggest that PPARβ/δ was causally related to cancer was the report that expression of PPARβ/δ could be increased by adenomatous polyposis coli (APC)/β-CATENIN/T-cell factor-4 (TCF4) signaling in colon cancer.^25^ This study was based on a number of experimental approaches including the demonstration that overexpression of APC caused downregulation of PPARβ/δ in a human colon cancer cell line, that relative expression of *PPARB/D* mRNA in four human colorectal cancer patients was higher in colon tumors, whereas expression of *PPARB/D* mRNA was essentially absent in normal colon epithelium, and that knocking down TCF4 a downstream transcription factor that mediates APC/β-CATENIN signaling in colon cancer mitigates expression of *PPARB/D* mRNA in three human colon cancer cell lines.^25^ A subsequent study by this same research group also suggested that deletion of expression of PPARβ/δ in two clonal variants of a human colon cancer cell line markedly reduced ectopic xenograft growth as compared with controls, but that this effect was not influenced by inhibition of cyclooxygenase activity^26^ as suggested previously.^25^

Some studies support the notion that expression of PPARβ/δ is higher in colon tumors as compared with control tissue. For example, northern blot analyses of six rat or human colon tumors and normal tissue suggested that expression of *PPARB/D* mRNA was negligible in non-transformed tissue but higher in paired colon tumors of both species.^27^ Similarly, relative expression of *PPARB/D* mRNA in 12 human patients was reportedly higher as compared with control tissue based on qPCR analyses.^28^ Relative expression of PPARβ/δ protein was examined in APC^*min/+*^ mouse colon tumors using IHC and western blot analyses from a small cohort (≤ 8 mice).^29^ In these studies, it was suggested that the relative expression of PPARβ/δ was higher in flat, dysplastic adenomas from APC^*min/+*^ mice as compared with non-transformed tissue, but this analyses was based on an unknown number of samples examined by IHC (see below).^29^ Importantly, western blot analyses did not support the expression of PPARβ/δ detected by IHC in these studies.^29^ Most recently, expression of *PPARB/D* mRNA was reported to be higher in tumors from twenty-two colon cancer patients as compared with control tissue, and this observation was consistent with IHC analyses of 152 colon tumors and non-transformed tissue.^30^ There are limitations with these studies^25–30^ because not all examined protein expression, and protein analyses utilized IHC in most cases, and the required controls validating the antibody used for IHC and the interpretations were not provided or described. Most of these studies also relied solely on mRNA analyses and not all performed quantitative analyses of *PPARB/D* mRNA expression. Importantly, these studies were inconsistent with the fact that basal expression of PPARβ/δ protein is relatively high in colon tissue. Thus, the original hypothesis that expression of PPARβ/δ is increased by APC/β-CATENIN/TCF4 signaling in colon cancer is not supported by many subsequent studies.

To more directly test the hypothesis that PPARβ/δ is increased by the APC/β-CATENIN/TCF4 signaling in colon cancer, relative expression of PPARβ/δ was determined using quantitative western blot analyses in a panel of human colon cancer cell lines including a cell line that had wild-type *APC* and *CTNNB1* genes (RKO cells), as well as cell lines with mutant *APC* and/or *CTNNB1* (DLD1, HCT-116, HT29, or LS174T cells).^31^ Although expression of the well-characterized APC target CYCLIN D1 was markedly higher in the cell lines with mutant *APC* and/or *CTNNB1* this was not observed in the control RKO cell line.^31^ These analyses included a positive control for PPARβ/δ and were based on statistical analyses of at least three independent biological replicates per cell line. Examination of PPARβ/δ was also examined in normal intestinal tissue and compared with PPARβ/δ expression in tumors in both mouse and human models. Three independent studies revealed that the relative expression of PPARβ/δ protein and/or mRNA was decreased in APC^*min/+*^ mouse or human colon tumors.^32–34^ One study showed that relative expression of *Pparb/d* mRNA was lower in colon tumors from APC^*min/+*^ mice as compared with non-transformed colon tissue (*n* = 12).^34^ Similarly, two additional studies revealed that the relative expression of PPARβ/δ protein was lower in colon tumors from APC^*min/+*^ mice as compared with non-transformed colon tissue.^32,33^ Importantly, one of these studies included a positive control for PPARβ/δ in the western blot, and also showed that the relative expression of CYCLIN D1 was markedly higher in colon tumors from APC^*min/+*^ mice as compared with non-transformed colon tissue.^33^ In human familial adenomatous polyposis (FAP) patients, the relative expression of *PPARB/D* mRNA was lower in colon tumors (*n* = 12 FAP patients) as compared with non-transformed colon tissue, but the *p* value was only 0.083.^34^ Similarly, the relative expression of *PPARB/D* mRNA was lower in colon tumors (*n* = 19 human colon cancer patients) as compared with non-transformed colon tissue.^33^ Moreover, quantitative analyses of protein revealed that whereas expression of CYCLIN D1 was increased in tumors from human colon cancer patients (*n* = 19), the relative expression of PPARβ/δ was lower in tumors as compared with control non-transformed tissue.^33^ This is also consistent with data from the HPA that examined a cohort of human colon cancer patients and reported that relative expression was lower in colon tumors as compared with non-transformed tissue.^16–18^

To further examine this putative signaling, several other approaches have recently been undertaken. In a human cell line that has a mutant *APC* gene (HT29), the expression of wild-type APC was reintroduced and expression of PPARβ/δ was examined. Interestingly, relative expression of *PPARB/D* mRNA was lower in control HT29 cells compared with wild-type HT29 cells with forced expression of APC (*p* = 0.062, *n* = 6 samples).^34^ This suggests that mutant APC might actually decrease expression of PPARβ/δ. The role of APC and downstream signaling proteins (β-CATENIN/TCF4) have also been examined using global gene expression profiling. Using the Cancer Omics Atlas,^35^ a web-based tool that performs analyses of the Cancer Genome Atlas (TCGA) database (https://www.cancer.gov/about-nci/organization/ccg/research/structural-genomics/tcga), relative expression of *PPARB/D* mRNA is lower (*p* = 0.009) in human colon tumors (*n* = 286) as compared with control tissue (*n* = 41). Analyses of data sets available in the Gene Expression Omnibus (GEO) also provide convincing evidence that β-CATENIN/TCF4 signaling does not increase expression of *PPARB/D* mRNA in colorectal cancer models, both in vivo and in vitro, including those with mutant *APC* or *CTNNB1* genes. For example, DLD1 and LS174T human colon cancer cell lines expressing a dominant negative TCF4 exhibit cell cycle arrest (G1) that is associated with decreased expression of the β-CATENIN/TCF4 target gene *MYC*, but an increase in expression of *PPARB/D* mRNA (GSE3282).^36^ Similarly, changes in gene expression caused by a dominant-negative TCF4 or silencing β-CATENIN expression in human colon cancer cell lines include decreased expression of known β-CATENIN/TCF4 target genes including *CCND1* and *AXIN2*, but an increase in expression of *PPARB/D* mRNA (GSE18560, GSE46465).^37,38^ By contrast, in human and rodent tumors expression of *CCND1* is increased, whereas expression of *PPARB/D* mRNA is decreased as compared with normal tissue (GSE68468, GSE41258).^39^ It is interesting that data from these studies actually suggest that β-CATENIN/TCF4 may repress expression of PPARβ/δ. Combined, these studies collectively do not support the hypothesis that expression of PPARβ/δ is increased by APC/β-CATENIN/TCF4 signaling in colon cancer, as both the mouse and human models examined include those with defective APC signaling. The studies showing that the relative expression of PPARβ/δ was lower in colon tumors as compared with non-transformed tissue provide strong weight of evidence for the field because they included larger sample sizes, included positive and negative controls, quantitatively examined protein expression, are consistent with large, publicly available databases, and directly compared models with defective APC signaling.

It is also worth noting that an alternative regulatory role for PPARβ/δ in colon carcinogenesis was subsequently hypothesized that was co-dependent on the administration of non-steroidal anti-inflammatory drugs (NSAIDs). It is accepted that NSAID administration can inhibit colon carcinogenesis, at least in part, by decreasing the production of cyclooxygenase (COX)-derived metabolites that function to promote cell proliferation and other mechanisms required for tumor progression.^40^ It was also suggested that NSAIDs may inhibit colon carcinogenesis by downregulating expression of PPARβ/δ as observed from in vitro and in vivo studies.^41,42^ However, follow-up studies by other laboratories were unable to reproduce these findings^31,43^ (reviewed in refs ^10,44–48^). In one example, the identical samples from a study suggesting that an NSAID inhibited expression of PPARβ/δ, which mediated inhibition of colon tumorigenesis were used in a collaborative effort to examine expression of PPARβ/δ in greater detail. In this latter study, it was found that although IHC analyses detected lower PPARβ/δ expression in colon tumors from mice treated with an NSAID,^41^ quantitative western blot analyses revealed that there was in fact no change in relative expression of PPARβ/δ between control and NSAID-treated colon tumors (or non-transformed tissue).^31^ Additional studies based on dose-dependent analyses of NSAID-induced inhibition of human colon cancer cell lines also revealed no decrease in expression of PPARβ/δ by NSAIDs at concentrations that inhibited cell proliferation of these colon cancer cell lines. In fact, one NSAID actually increased expression of PPARβ/δ in RKO cells, consistent with other studies using different models^31^ (reviewed in refs ^10,44–48^). This illustrates two important points: (1) downregulation of PPARβ/δ expression does not appear to be a mechanism by which NSAIDs inhibit colon cancer, and (2) IHC analyses of PPARβ/δ expression can be misleading, most likely influenced by non-specific binding of anti-PPARβ/δ antibodies. The latter is relevant to many studies that also relied on IHC without all of the required controls to assess PPARβ/δ expression in colon cancer models (reviewed in refs ^10,44–48^).

The weight of evidence indicates that relative expression of PPARβ/δ is not upregulated by APC/β-CATENIN/TCF4 signaling in colon cancer. This is consistent with the changes made by the National Center for Biotechnology Information (NCBI) for the description of human PPARβ/δ. In 2010 the NCBI website listed the following for the description of PPARβ/δ: “*………….PPARs mediate a variety of biological processes, and may be involved in the development of several chronic diseases, including diabetes, obesity, atherosclerosis, and cancer. This protein is a potent inhibitor of ligand-induced transcription activity of PPAR alpha and PPAR gamma. …………The expression of this gene is found to be elevated in colorectal cancer cells. The elevated expression can be repressed by adenomatosis polyposis coli (APC), a tumor suppressor protein related to APC/beta-catenin signaling pathway. …………*. [provided by RefSeq, Jan 2010]”. However, in 2017 this was changed to: “*………..The encoded protein is thought to function as an integrator of transcriptional repression and nuclear receptor signaling. It may inhibit the ligand-induced transcriptional activity of peroxisome proliferator-activated receptors alpha and gamma, though evidence for this effect is inconsistent. Expression of this gene in colorectal cancer cells may be variable but is typically relatively low. ……………*.. [provided by RefSeq, Aug 2017]. Note that the reference to APC-regulating PPARβ/δ, and the suggestion that relative expression is higher in colorectal cancer cell are no longer included in the NBCI description. This is an excellent example of how our understanding of the regulation of PPARβ/δ in colon cancer has evolved, and it is now accepted by a stronger weight of evidence that expression of PPARβ/δ is typically lower in colon cancer.

Because of the confusion about the fundamental level of PPARβ/δ expression in colon cancer, many questions have surfaced about the role of PPARβ/δ in colon carcinogenesis. In particular, does relative expression of PPARβ/δ change during tumor progression and if so, does the change in expression modulate tumorigenesis? Although questions are co-dependent on the potential role of endogenous ligands of PPARβ/δ, which are known to exist and cause chronic, PPARβ/δ activity in many mammalian cells, the focus of the remainder of this review is on the relative expression of PPARβ/δ in cancer and what has been learned in the past 20 years, with an emphasis on colon cancer. Owing to the scope of this review, the remainder of the review will only provide limited insight into the effects of exogenous and endogenous ligands and potential mechanisms mediated by PPARβ/δ on colon cancer.

## Mouse and human loss-of-function PPARβ/δ models of colon cancer

The first study to examine the potential role of PPARβ/δ in a colon cancer model suggested that PPARβ/δ was dispensable for colon carcinogenesis as no differences in tumor multiplicity or size was observed between control APC^*min/+*^ mice and APC^*min/+*^ mice crossed with a *Pparb/d*-null mouse line.^49^ However, only three double mutant mice on a mixed genetic background were examined in this study. This study is limited because of the small sample size, and the fact that the genetic background of mice can markedly influence the outcome of a colon cancer bioassay, in particular because of known modifier of Min genes that can be present in mice with a mixed genetic background.^50–52^ Genetic disruption of PPARβ/δ expression also had no effect on intestinal tumorigenesis in APC^*min/+*^ mice, following administration of azoxymethane/dextran sulfate sodium (AOM/DSS), or in a mutant *Pparb/d*-null mouse line with a disrupted mismatch repair gene (*Mlh1*) gene.^53–55^ Interestingly, tissue-specific disruption of PPARβ/δ expression in colonocytes also did not influence AOM/DSS-induced colon tumor multiplicity in mice fed semi-purified diets containing either fatty acids thought to promote or inhibit colon carcinogenesis.^56^ By contrast, a more rigorous study using much larger sample sizes and congenic mice demonstrated that colon carcinogenesis is exacerbated in the absence of PPARβ/δ expression in both a genetic (APC^*min/+*^ mice crossed with *Pparb/d*-null mice) and a chemically-induced model AOM.^57^ The latter study is supported by other investigations using the same mouse line that showed that colon carcinogenesis is exacerbated in the absence of PPARβ/δ expression.^58,59^ There are two studies published to date suggesting that genetic disruption of PPARβ/δ expression mitigates colon tumorigenesis in APC^*min/+*^ mice.^60,61^ In the first study, colon tumor multiplicity was ~ 2 polyps per control APC^*min/+*^ mice as compared with ~ 1 polyp per APC^*min/+*^ X *Pparb/d*-null double mutant mice in both males and females.^60^ However, in the same study but a different experiment, colon tumor multiplicity was ~ 1 polyp in both control APC^*min/+*^ mice and APC^*min/+*^ X *Pparb/d*-null double mutant mice in both male and female mice.^60^ Although total intestinal tumor multiplicity was lower in male and female APC^*min/+*^ X *Pparb/d*-null double mutant mice as compared with control APC^*min/+*^ mice, the disparity in colon tumorigenesis could be influenced by genetics because the mice used for these studies were of mixed genetic background (Sv/129 X C57BL/6), which is known to cause significant variation in colon cancer bioassays.^50–52^ A second study examined AOM-induced colon tumorigenesis using congenic *Pparb/d*-null mice and an intestine-specific PPARβ/δ disruption approach.^61^ AOM-induced colon tumorigenesis was essentially mitigated in mice expressing villin-Cre to disrupt PPARβ/δ expression in the colonocytes as compared with *Pparb/d* wild-type mice.^61^ However, there are significant issues with this study. First, AOM-induced colon tumorigenesis was decreased in mice harboring the flanking LoxP sites surrounding the *Pparb/d* alleles but without Cre expression.^61^ Thus, expression of PPARβ/δ was not lost in the intestines of these mice but the same phenotype was observed in the mice with colonocyte-specific loss-of PPARβ/δ. Moreover, the *Pparb/d*-null mice used for these studies also revealed no difference in colon tumorigenesis in previous studies using either the APC^*min/+*^ mouse line or the AOM-induced colon cancer model, and the latter model utilized an identical intestinal-specific PPARβ/δ disruption approach.^54,55^ Although this latter disparity could be reflected by strain differences, the lack of reproducibility with related models raises concerns about this study.

These collective in vivo studies have yielded conflicting results about the role of PPARβ/δ expression in colon cancer and it is tempting to speculate that differences in the null mouse models could underlie, at least in part, the distinct phenotypes. However, there is consistent evidence that PPARβ/δ attenuates epidermal cell proliferation from three independent laboratories^62–65^ using the same three *Pparb/d*-null mouse lines used for the above studies. Further studies are needed to determine the reasons for the differences in the results obtained from simple genetic disruption of PPARβ/δ expression using in vivo colon cancer models.

Knockdown of PPARβ/δ expression in human colon cancer cell lines in vitro has also been used to examine the role of this receptor in colon cancer models. Knockdown of PPARβ/δ expression in HCT-116 human colon cancer cells caused enhanced cell proliferation owing to a block in the G1 phase of the cell cycle with no changes in apoptosis as compared with controls.^66^ Consistent with these observations, knockdown of PPARβ/δ expression in KM12C, KM12SM, and KM12L4a cell lines caused enhanced proliferation, and a more malignant morphology of clonal colonies that was associated with reduced differentiation and enhanced cell-fibronectin adhesion of these cell lines as compared with controls.^67,68^ This suggests that basal PPARβ/δ expression in colon cancer cell lines is associated with attenuating proliferation and reduced metastatic potential. Using these cell lines, this group also demonstrated that knockdown of PPARβ/δ expression in KM12C human colon cancer cell lines markedly enhanced ectopic xenografts derived from these cells that was also associated with increased cell proliferation, reduced differentiation, enhanced expression of vascular endothelial growth factor (VEGF) as compared with controls.^69^ The latter effect appears to be related to the reduced sensitivity of the xenografts derived from KM12C PPARβ/δ knockdown cells to the VEGF inhibitor bevacizumab observed in these studies.

## Mouse gain-of-function PPARβ/δ model of colon cancer

Forced expression of PPARβ/δ in colonocytes by use of the villin promoter increased colon tumor multiplicity in two strains of the PPARβ/δ transgenic mice as compared with controls.^70^ Although these studies are of interest, it is important to note that transgenes are known to become incorporated into the genome in multiple sites and in tandem repeats and can disrupt genes near the site of integration. Thus, despite the fact that more than one line was examined in these studies, it remains possible that the phenotype observed is due in part to potential influence on genes neighboring the *Pparb/d* integration site(s).

## Correlative analyses of PPARβ/δ expression in colon cancer

**Fig. 2:**
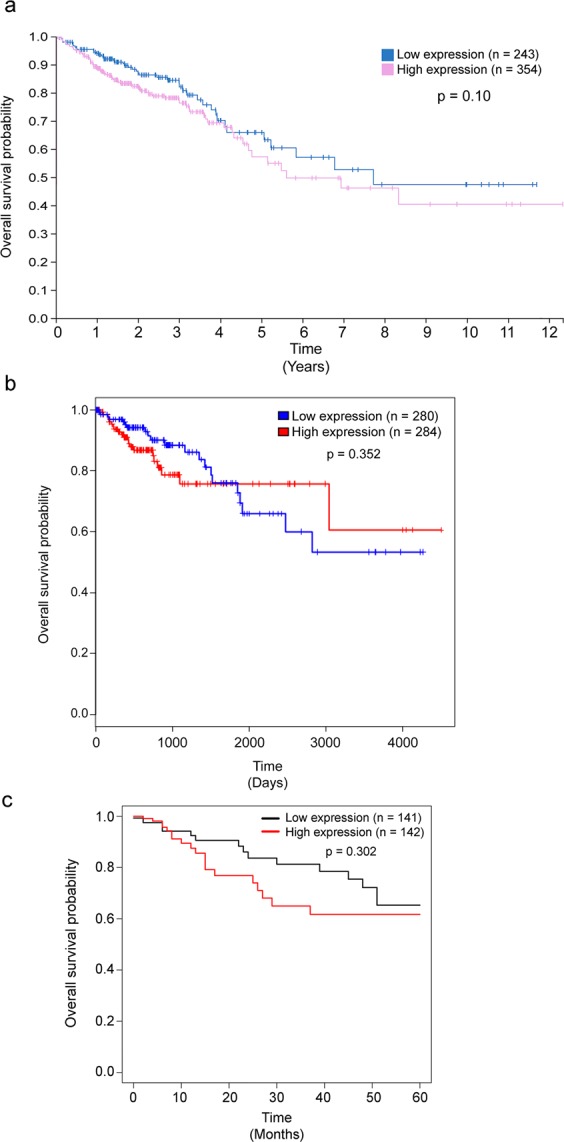
Kaplan–Meier plots of relative expression of *PPARB/D* mRNA and human colon cancer patients. **a** Analyses from the Human Protein Atlas.^16–18,81^ This analysis is based on the Fragments Per Kilobase of transcript per Million (FPKM) mapped reads value of each gene; patients were classified into two expression groups (low or high) and the correlation between *PPARB/D* mRNA expression level and patient survival was examined. Genes with a median expression less than FPKM 1 were excluded. The prognosis of each group of patients was examined by Kaplan–Meier survival estimators, and the survival outcomes of the two groups were compared by log-rank tests. The median separated Kaplan–Meier plots are presented in the Human Protein Atlas figure, and genes with log-rank *P* values < 0.001 in maximally separated Kaplan–Meier analysis were defined as prognostic genes. If the group of patients with high expression of a selected prognostic gene has a higher observed event than expected event, it is an unfavorable prognostic gene, otherwise, it is a favorable prognostic gene. Based on this analysis relative expression of *PPARB/D* mRNA is not prognostic for colorectal cancer. **b** Kaplan–Meier survival plots generated from The Cancer Omics Atlas comparing data from the TCGA database.^35,82^ Relative expression below the median values were grouped as low and median values above the median grouped as high. No differences in overall survival were determined between colorectal cancer patients with low or high expression of *PPARB/D* mRNA. **c** Kaplan–Meier survival plots generated by comparing data from the TCGA database.^82^ Relative expression below the median values were grouped as low and median values above the median grouped as high. No differences in overall survival were determined between colorectal cancer patients with low or high expression of *PPARB/D* mRNA

Correlative analyses between relative expression of intestinal PPARβ/δ mRNA or protein and colon cancer patient survival have also been undertaken to try and establish potential associations between the two variables. Analyses from the HPA, the Cancer Omics Atlas, and in house data revealed no correlation between overall patient survival and patients with relatively low or high *PPARB/D* mRNA (Fig. 2). Importantly, these analyses are based on sample sizes that are well powered (*n* = 141–354). By contrast, analyses of a small cohort (*n* = 66) of colorectal cancer patients showed a correlation between high expression of *PPARB/D* mRNA and lower survival as compared with colorectal cancer patients with low expression of *PPARB/D* mRNA.^30^ Because this latter analyses was limited to data from 33 patients in each group the study is not powered as strongly as the former analyses.

Correlative analyses of relative PPARβ/δ protein expression and survival of colorectal cancer patients have also been performed. In a very small cohort of patients, it was suggested that co-expression of PPARβ/δ and COX2 proteins was negatively associated with patient survival.^71^ However, this study relied on IHC for estimating relative expression of PPARβ/δ and as noted above, this methodology has many inherent problems with accurately quantifying protein expression not controlled for in this study. In addition, the analyses were limited to only 17 patients that exhibited co-expression of PPARβ/δ and COX2 proteins and 33 patients that exhibited unclear expression of either PPARβ/δ and COX2. Thus, the power of this study is limited for determining associations between PPARβ/δ and colon cancer patient survival. A correlative study focused on the relationship between PPARβ/δ protein expression in primary tumors and colorectal cancer patient survival was also performed revealing that colorectal cancer patients with relatively higher expression of PPARβ/δ in the primary tumor (*n* = 26) were ~ 4× less likely to die from the disease as compared with those patients with relatively lower expression of PPARβ/δ in the primary tumor (*n* = 38).^68^ This study was particularly stronger than other studies because the authors confirmed PPARβ/δ expression by western blot. However, results from this study are curious because they indicated that the average expression of PPARβ/δ protein increased in primary tumors as compared with non-transformed colon tissue.

Correlative studies such as those described above have limitations that must be considered. First, these studies do not demonstrate cause and effect and require large scale experiments to draw a definitive causal relationship. Kaplan–Meier curves also have many limitations including the fact that they are used in univariate analyses that do not allow for control of co-variates that could markedly impact the interpretation and provide no estimate of effect size.^72–74^ Moreover, changes in *PPARB/D* mRNA levels could merely reflect effects or compensatory changes due to factors related to the disease^47^ (see below). In addition, it is important to point out that mRNA levels of genes do not always correlate with actual protein expression and as such, the Kaplan–Meier curves derived by this method have this inherent limitation as well. The fact remains, the most definitive approach to determine the role of PPARβ/δ in human cancer would be to perform prospective or retrospective studies with humans treated with PPARβ/δ agonists, and these data do not exist in the public database. That withstanding, it is worth noting that there are many studies that have examined the effect of expression and/or ligand activation of PPARβ/δ in animal/human cancer models. Unfortunately, these studies are highly contradictory (reviewed in refs ^10,44–48,75,76^).

## Conclusions and future directions

It is clear from extensive, rigorous analyses using multiple approaches that the original hypothesis that the mutant APC pathway upregulates expression of PPARβ/δ in colon cancer cells^25^ is not supported by the weight of evidence. Future studies should recognize the review of this literature and acknowledge the studies that provide this strong weight of evidence, in particular those derived from unbiased, large databases. Indeed, the current weight of evidence indicates that expression of PPARβ/δ at both the mRNA and protein levels is typically lower in colon tumors in both animal models and humans as compared with control tissue. It is clear from this review of the literature that there remains a need to reconcile the opposing hypotheses of the role of PPARβ/δ in colon carcinogenesis, especially as basal expression of PPARβ/δ is known to be very high in normal colon. Colon cancer is not prevalent in a high percentage of humans and the average lifetime risk of developing this disease is ~4% in the US population in the absence of major risk factors.^77^ These facts are inconsistent with a protein that is expressed at high levels and is constitutively active if it were involved in the promotion of colon cancer. In addition, mutations in the *PPARB/D* gene are extremely rare (≤1.4%) in colorectal cancer patients based on analyses of TCGA data using the Cancer Omics Atlas.^35^ This is in contrast to the high frequency of mutations in genes such as *TP53* (~59%), *KRAS* (45%), or *APC* (82%) that are known to be key driver mutations in colorectal cancer.^78^ This suggests that mutations in the *PPARB/D* gene that could produce mutant protein are unlikely to contribute to colon cancer. Moreover, although not addressed in this review, what is/are the natural agonist(s) or antagonist that modulate PPARβ/δ activity to promote colon cancer as hypothesized by some? Given that PPARβ/δ expression is naturally high in the colon, gene mutations in *PPARB/D* occur with very low frequency in colon tumors, and expression levels of *PPARB/D* mRNA are not associated with colon cancer patient survival, it makes much more sense that natural agonists or antagonists function to maintain homeostasis in colon cells by promoting terminal differentiation or attenuating inflammation. Future studies should be focused on these ideas.

There are examples where expression of PPARβ/δ mRNA and/or protein may be increased in colon tumors compared with control tissue, and this may reflect a compensatory change as hypothesized >10 years ago.^47^ Expression of PPARβ/δ can be increased by numerous stimuli most notably molecules that increase inflammation or stress (tumor necrosis factor-α, interferon-γ, phorbol ester, lipopolysaccharide, etc.) most likely owing to the presence of an AP1 regulatory site in the promoter region of the *PPARB/D* gene.^79,80^ Thus, increased expression of PPARβ/δ in these circumstances could be a protective mechanism by which the cell is able to resolve inflammation and/or promote terminal differentiation. For this reason, the relatively higher expression of PPARβ/δ that are inconsistently observed in some studies in colon tumors could be mediated by this mechanism but should be not viewed as being causally related to promoting colon carcinogenesis.

The evidence suggesting that NSAIDs decrease expression of PPARβ/δ in colon cancer cells is also not supported by the weight of currently available evidence (reviewed in detail in ref. ^48^). It is of interest to note that NSAIDs can decrease the production of bioactive molecules that could increase expression of PPARβ/δ due to their ability to promote inflammation. However, most NSAIDs fail to influence expression of PPARβ/δ in human colon cancer cells at concentrations that cause inhibition of proliferation.^31^ As inflammation is common in cancer but not present in all cases, the lack of change in expression of PPARβ/δ by NSAIDs may reflect the lack of induced expression of COX2 in some colorectal cancer patients.

Although not in the scope of the present review, there are clearly contradictory results reported for the effects of synthetic PPARβ/δ agonists in cancer models including colon cancer. The effects observed in cancer models, resulting from the administration of synthetic PPARβ/δ agonists need to be reconciled with the effects of natural ligands and their ability to mediate important physiological functions. Finally, one way to help clarify the role of PPARβ/δ expression and molecules that modulate PPARβ/δ activity would be to encourage collaborative efforts between laboratories reporting the conflicting results. Indeed, this approach has proven useful in the past.^33,41^ As PPARβ/δ is a nodal transcription factor that regulates numerous genes, delineating how to best target this protein in colon cancer and other cancers will likely yield a solid approach for both cancer chemoprevention and chemotherapy.

## Supplementary information

ProteinAtlastUsage.pdf

ReportingSummary

## Data Availability

The data used to create Fig. 1a, b were obtained from the HPA.^16–18,81^ Relative expression of protein or mRNA based on analysis from the HPAs on September 15, 2019, Version 19, Ensembl version 92.38. The data used to create Fig. 2a–c were obtained from the TCGA project based in part using the web-based Cancer Omics Atlas analyses; using the free, publicly available TCGA databases or a free, publicly available web-based analytical platform, respectively.^35,82^
