# Supplementary material for: Unraveling the role of peroxisome proliferator-activated receptor-β/δ (PPARβ/δ) expression in colon carcinogenesis
Source: NPJ Precis Oncol. 2019 Oct 7;3:26. doi: 10.1038/s41698-019-0098-x (PMC6779880; doi:10.1038/s41698-019-0098-x)
Supplement: Supplementary file 1 — ProteinAtlastUsage.pdf [file 41698_2019_98_MOESM1_ESM.pdf]

Subject: SV: question about usage of Protein Atlas in publication
From: "contact@proteinatlas.org" <contact@proteinatlas.org>
Date: 1/21/15, 10:45 AM
To: Jeff Peters <jmp21@psu.edu>

Dear Jeff,

We are glad to see that you are using the protein atlas.
Since you already are clear on how to handle the citations you are welcome to use the data.
No, publications are not viewd as commercial, i.e. no problem to go ahead with your submission.

good luck with the publication.

Best regards
Evelina Sjöstedt
the Human Protein Atlas
Uppsala University

Från: Jeff Peters [jmp21@psu.edu]
Skickat: den 20 januari 2015 16:02
Till: contact@proteinatlas.org
Ämne: question about usage of Protein Atlas in publication

To Whom It May Concern,

I have a question regarding a Figure I made from the data provided on your fantastic website. I have cited your website and the manuscripts you point out when I refer to these data in other previously published work from my lab.

However, I wanted to make sure I was conforming to your guidelines before publication. I cite (www.proteinatlas.org<http://www.proteinatlas.org>), I also cite the Uhlen references, and I refer to the version of the

website that I used.

Since journals charge for subscriptions, would the inclusion of a Figure I made from your data represent a "commercial" use, or would fall under "informational" and be acceptable? Do I need to obtain permission from you for this?

Thanks in advance for your help.

Sincerely,

Jeff
--
Jeffrey M. Peters, Ph.D., A.T.S.
Distinguished Professor
Center for Molecular Toxicology and Carcinogenesis
Department of Veterinary and Biomedical Sciences
312 Life Sciences Building
The Pennsylvania State University
University Park, PA 16802
(814) 863-1387
(814) 863-1696 FAX
jmp21@psu.edu<mailto:jmp21@psu.edu>
http://www.cmtc.psu.edu/peters_group/peters_home.asp
